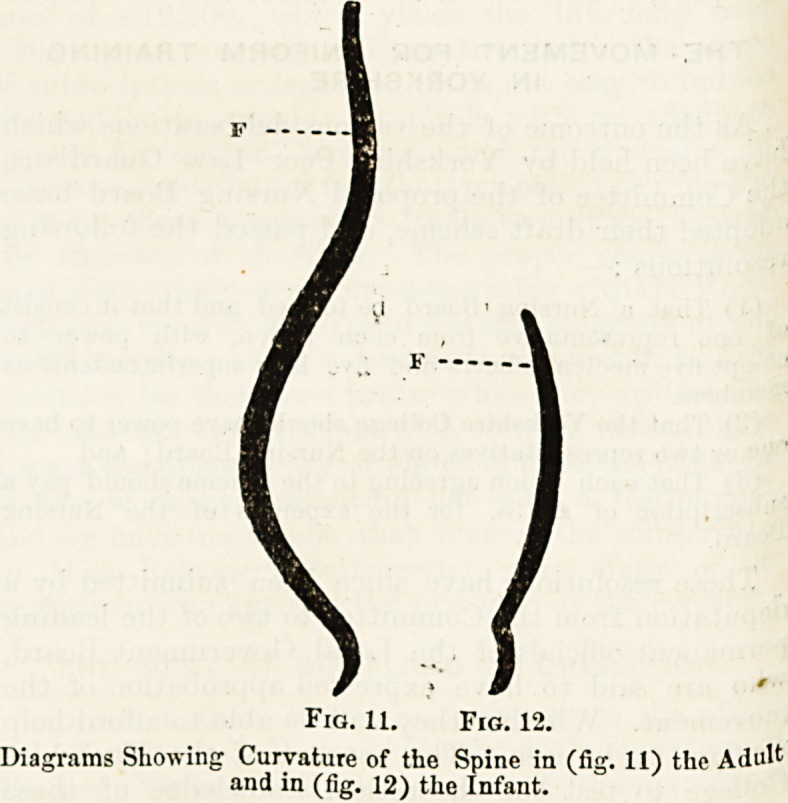# The Hospital. Nursing Section

**Published:** 1901-11-30

**Authors:** 


### The Hospital.

### Nursing Section.

Contributions for this Section of "The Hospital" should be addressed to the Editor, "The Hospital" Nursing Section, 28 & 29 Southampton Street, Strand, London, W.C.

No. 792.—Vol. XXXI. *SATURDAY, NOVEMBER* 30, 1901.

### Notes on News from the Nursing World.

### OUR CLOTHING DISTRIBUTION.

We have to acknowledge with many thanks the receipt of parcels of useful clothing as follows:—No. 37, The Wilderness, Mitcheldean, Gloucestershire; Nurse S. A. Mallinson, 93 Onslow Gardens, S.W.; Nurse Aldridge, The Gables, Ipswich; Evelyn M. Smith, 3 Mortimer Villas, High Road, North Finchley; and Policy 1081, 13 Redesdale Street, Tedworth Square, Chelsea. We again remind our numerous friends who intend to assist us to provide acceptable Christmas gifts for patients in hospitals and infirmaries that the latest day to send in parcels is Monday, December 16. The name and address of the sender should be enclosed in each parcel, which should be directed to the Editor of the Hospital, 28 & 29 Southampton Street, Strand, London, W.C., marked "Clothing Distribution."

### PENSION FUND SOUVENIR.

The proprietors of The Hospital have been asked whether there will be space in the Souvenir Album for the inclusion of a Policy Certificate. They are able to say that the Album is large enough for this purpose, and that there is a blank page at the end of the book which might be suitably utilised. The idea is a practical one, as not all nurses can carry a framed certificate with them, whereas in the Album it is portable and appropriately placed.

### THE SOUTH AFRICAN CONSTABULARY.

In consequence of the publicity given in our columns some weeks ago to the conditions of service in the nursing establishment of the South African Constabulary, there have been numerous applications, and the High Commissioner for South Africa is anxious it should be known as widely as possible in the nursing world that there are no vacancies now open. Eligible candidates who have offered themselves for appointment may, however, rest assured that in the event of vacancies arising, or of further help being required, they will be communicated with.

### THE NEEDS OF THE SMALL COLONIES.

The speech of Mr. Chamberlain on behalf of the Scottish Branch of the Colonial Nursing Association has had a stimulating effect on the other side of the Border, and public meetings in aid of it are to be held at Glasgow, Dundee, Perth, and Aberdeen. The total subscriptions and fees received are between £300 and £400, and it is significant that most of this amount has been contributed by the people of Edinburgh, many of whom had the pleasure of hearing the appeal of the Colonial Secretary. It is hoped that the balance required—only £1,000 is asked for—will be collected before Christmas. Meanwhile, we observe that Mr. David Richmond has wisely been emphasising the fact that the fund is intended only for the benefit of the smaller and poorer colonies and dependencies of the Empire. This cannot be too clearly and persistently pointed out. It was not in the interests of the large and wealthy colonies that Mrs. Frances Piggott originated the organisation. As our readers will remember, the undertaking owes its existence to her desire that the Crown colonies, which, as at Mauritius, had not seven years ago an English trained nurse available for love or for money, should be able to command services which are so highly valued at home as well as in those parts of the Empire beyond the seas where they are easily obtainable.

### "GUY'S" AND THE CONCENTRATION CAMPS.

The four sisters from Guy's Hospital, whose conditional appointment as matrons of the Concentration Camps for Boer women and children was announced in The Hospital last week, have been passed by the medical adviser of the Colonial Department, and will start next week for South Africa. Their selection by Mr. Chamberlain is a great compliment to Guy's as an institution, and we congratulate not only Sisters Willes, Jones, Hyland, and Fennimore upon being chosen for work of a very delicate and responsible character, but also Miss Swift, the matron, upon the fact that the Colonial Secretary has expressed his gratitude for the trouble she has taken in recommending the candidates.

### THE WAR NURSES.

The *Roslin Castle* arrived at Southampton on Sunday (24th) from South Africa, and the following nursing sisters disembarked:—E. A. L. Fry, A.N.S.R., requires one month's leave and returns to South Africa; J. Joel, A.N.S.R., requires six months' leave and passage back to South Africa; A. McKillen, A.N.S.R., requires 42 days' leave and passage to New Zealand; M. Nicholson, A.N.S.R., requires 42 days' leave and returns to South Africa; A. Peiper, New Zealand, N.S., requires 42 days' leave and passage to New Zealand; J. W. M. Williamson, New Zealand, N.S., requires 42 days' leave and passage to New Zealand. Two sisters returned as invalids, namely, S. E. Oram, A.N.S., superintendent sister, requires three months' sick leave and returns to South Africa; and A. Gore, A.N.S.R., requires three months' sick leave.

### TESTIMONIAL TO MISS AMY HUGHES.

On Wednesday afternoon a very interesting ceremony will take place at the Portland Hotel, Great Portland Street. The Nurses' Co-operation will be "at Home" from 3 to 5 "to meet Miss Hughes," who is to be presented with a testimonial consisting of a diamond ring and a watch. The testimonial is in recognition of the valuable services of Miss Hughes as lady superintendent of the Nurses' Co-operation, and the nurses of "the Co." will doubtless muster in force in order to witness the function, and to cheer the popular recipient of the gifts. The ring is set with diamonds, and the watch, which is gold and enamel, has the inscription on the inner case, "Presented to Miss Hughes by the Nurses of the Co-operation, 1901."

### POOR LAW GUARDIANS AND DISTRICT NURSING.

Both at Sheffield and at Hereford a new organised attempt is being made to provide nurses for sick paupers in their own homes. At Hereford thé idea is that the Rural District Council should engage nurses for infectious cases to be nursed in cottages rented by them, and that when there are no cases of the kind requiring attention, the Hereford Board of Guardians should make use of the nurses, either at the Workhouse Infirmary or the Isolation Hospital. As to financial arrangements, it is suggested that each body should pay the salaries of the nurses during the time they employed them. But supposing that, when there were no infectious cases, the Board of Guardians did not want any additional nurses? This point will, we presume, be raised next month, when the scheme is to be fully considered by the District Council. The project at Sheffield is of a more far-reaching character, and, briefly stated, aims at the nursing of paupers in their own homes by the nurses employed under the Poor Law Act. The sub-committee of the Sheffield Board of Guardians have definitely recommended the appointment of four district nurses, at a salary of £75 each, to reside in or near to their district, to receive orders through the relieving officer, and to be subject to the control of a special committee of five guardians. During the course of a long discussion one of the guardians inquired whether these nurses would be available for a working man getting 21s. or 25s. per week who had sickness in his family, and the Chairman accordingly replied that "the law did not give them power to provide for anyone except paupers." In the end it was decided to defer the matter for six months in order to obtain all possible information from other Unions.

### A SUGGESTION TO THE PRESIDENT OF THE LOCAL GOVERNMENT BOARD.

Miss Louisa Twining comes forward with the proposition that, following the example of the War Office in respect to the Army Medical Department, the Local Government Board should appoint a matron-in-chief, and three assistant matrons to assist the male members of the Board in the discharge of their duties. Miss Twining says: "When the subject of nursing the sick has become the chief and most important of the duties of the Poor Law, I venture to ask if it is unreasonable to demand that at least some women should be consulted on matters which have always been considered their especial province? I have long urged an increase of qualified women inspectors, but I add this request as one still more urgent and far-reaching." In view of the very meagre attention given by most of the inspectors of the Local Government Board to nursing in English workhouse infirmaries, as well as for other reasons, Miss Twining's suggestion merits Mr. Walter Long's consideration.

### ROYAL BRITISH NURSES' ASSOCIATION.

The annual conversazione of the Royal British Nurses' Association will be held under the patronage of the president, H.R.H. Princess Christian, at the Kensington Town Hall on Tuesday, December 3rd, at 8 p.m. The following artistes have kindly promised their assistance:—Miss Louie Heath, A.R.C.M., Miss Helen McCarthy, Miss Annie McDonnell, Mr. Stepney Rawson, and Mr. Norman Ridley. Miss Annie Esmond and Mr. David James will appear in the one-act comedy, "He and She." Tickets can be obtained from the Secretary, 10 Orchard Street, W. The sessional lecture of the Association will be given by Dr. Jane Walker on "The Open-Air Treatment of Consumption," at 10 Orchard Street, on Monday, December 2nd, at 5.30 p.m.

### A MATRON'S PENSION.

No one will grudge Miss Falconer the £35 a year which has been voted to her as a retiring allowance by the subscribers to the Stirling Royal Infirmary. Her nearly 30 years of service as matron were marked by unfailing devotion to her duties, and, in addition to her attention to the patients and her consideration for the nursing staff, she rendered practical assistance to the infirmary by personally and successfully exerting herself to obtain financial support. But we cannot blame the subscribers to the charity for mooting the general question whether pensions should be granted, though it certainly was not worth while to propose that the amount should be £20 instead of £35. Against the principle of giving pensions in a more or less haphazard manner according to the popularity of the recipient at the moment of her retirement, there is a good deal to be said. It was stated that the money for Miss Falconer's pension would not be taken "exactly from the contributions." But surely the invested sum of £10,000, which yields the infirmary over £300 a year, was originally contributed in the shape of subscriptions or legacies. It is not easy to induce the public to provide hospitals with an adequate income for general purposes, and the diversion of any sums contributed for that purpose, even for the most excellent purpose, is likely to militate against the interests of charities. The proper course is for each institution to work in co-operation with the Royal National Pension Fund, and for the members of the staff, from the matron downwards, to assist in making provision for their own future while they are earning full salaries. The friends of a retiring matron have always the opportunity of showing their appreciation of her work by presenting her with a testimonial, and we have no doubt that among the subscribers to Miss Falconer's testimonial were many of the gentlemen who opposed the principle of the pension.

### THE WEST OF SCOTLAND CO-OPERATION.

No fewer than 156 nurses are now on the roll of the Glasgow and West of Scotland Co-operation of Trained Nurses, and the object of the executive committee, as stated in last year's report, has therefore been achieved. Other interesting facts transpired at the annual meeting. The number of cases attended during the year ending September 30 was 1,296, as compared with 1,174 the previous twelve months. The gross sum earned by the nurses was £8,633, compared with £7,186. Two nurses earned £87 each; nine from £80 to £87; and the average over all for the year was about £70, allowing one month for holiday. The accounts showed that the total income was £1,046, and the expenditure £773, £197 of the surplus being transferred to the heritable property account and £76 carried forward. Since the opening of the war, eleven of the Co-operation nurses have volunteered for service, and all these are still in South Africa. In the course of an interesting speech Professor M'Call Anderson, who seconded the adoption of the report, said that when he compared the supply of nurses at the present time, both as to number and quality, with that which existed in the early period of his professional career, he was struck with the marvellous change for the better which had taken place. He testified to the assistance which the Co-operation was to the medical profession in Glasgow, because they knew that when they sent there they were almost sure to get what they wanted. The steady progress of the organisation certainly points to the conclusion that it is managed on business lines, and that the duties of the members are performed in a manner satisfactory to their patients.

### MUSIC-HALL ARTISTS AND QUEEN'S NURSES.

A music-hall paper suggests that Mrs. Adney Payne, who has collected £200 towards the Women's Memorial to Queen Victoria, would have done better to have used her "undoubtedly great influence" in aid of the Music-hall Benevolent Fund or the Home Fund. This is very ungracious, especially as music-hall singers, if sickness overtakes them in their rainy days, are often glad to obtain admission to hospitals and to derive benefit from the services of trained nurses. We congratulate Mrs. Adney Payne upon the excellent result of her effort to assist the memorial for the extension of the nursing movement among the sick poor, so dear to the heart of our late Sovereign.

### THE MOVEMENT FOR UNIFORM TRAINING IN YORKSHIRE.

As the outcome of the various deliberations which have been held by Yorkshire Poor Law Guardians, the Committee of the proposed Nursing Board have adopted their draft scheme, and passed the following resolutions :—

(1) That a Nursing Board be formed, and that it consist of one representative from each Union, with power to co-opt five medical officers and five lady superintendents as members.

(2) That the Yorkshire College should have power to have one or two representatives on the Nursing Board; and

(3) That each Union agreeing to the scheme should pay a subscription of £3 3s. for the expenses of the Nursing Board.

These resolutions have since been submitted by a deputation from the Committee to two of the leading permanent officials of the Local Government Board, who are said to have expressed approbation of the movement. Whether they will be able to afford help remains to be seen. The consent of the Yorkshire College to test the theoretical knowledge of these nurses whose practical efficiency is satisfactorily guaranteed by the Poor Law Unions, should be a real assistance in improving the nursing in Yorkshire workhouse infirmaries; but the College Medical Board have very properly made it a point that all nurses presented for such examination shall have had practical training for a certain minimum length of time in hospitals which reach a certain standard in regard to the number of beds, the variety of cases, and the methods of instruction.

### A NURSE SENT TO PRISON.

At the Devon Assizes, Clara Cooper, a nurse, was indicted for writing and publishing defamatory libels concerning Dr. C. N. Lovely, of Dawlish. She pleaded guilty. The evidence showed that she resorted to the device of writing anonymous libels to herself in order to divert suspicion. She was sentenced to six months' imprisonment, Mr. Justice Bruce stating that if it were found she was suffering from any mental disease she would be removed to an asylum.

### A BURGLAR IN A NURSE'S BEDROOM.

Exeter Sanatorium was entered by a burglar the other morning. He is supposed to have obtained ingress by the side door of the administration block, which is left open for the convenience of the night nurses. He ransacked the night nurses' bedrooms, taking from one room thirty-five shillings which was in a drawer. In one room a nurse was sleeping. Roused by the striking of a match she jumped up in bed, the man apologised for coming in, and said that she was wanted in the wards. He then left the room hurriedly. The nurse at once raised an alarm, but the man got away. It afterwards transpired that another burglary had been committed by the same man at a private house, a mile distant, two hours earlier. A burglar has since been arrested at Farnham, Kent, who answers to the description of the man seen in the vicinity of the Exeter Sanatorium the day previous to the burglary.

### ST. JOHN'S HOUSE NURSES.

The first general meeting of the League of St. John's House Nurses was held at St. John's House, Norfolk Street, Strand, on Saturday last. The chair was taken by the President, who opened the meeting with a few well-chosen words of welcome to all present. The bye-laws were fully discussed and adopted *en bloc*. Designs for badges were decided upon and other business was transacted. There are now 68 members of the League. The meeting was followed by a social gathering of old friends.

### SHORT ITEMS.

The second entertainment of the season for the in-patients of the Cancer Hospital, Fulham Road, was given by the Socials Dramatic Company on Thursday last. It consisted of three excellently-rendered short dramatic pieces and of violin and piano solos. The patients found the evening exceedingly enjoyable.—The training course for the certificate of the Sanitary Inspectors' Examination Board, under the auspices of the National Health Society, will begin in January, 1902, and will consist of at least 37 lectures and demonstrations.—At the invitation of the Society of American Women in London, the nurse delegates to the International Congress of Nurses at Buffalo will be entertained at a reception in the rooms of the society, at Prince's, on Friday, November 29th, at 3 p.m.—Miss K. M. Lumsden has, to the great regret of the directors, resigned the post of hon. superintendent of the Royal Aberdeen Hospital for Sick Children, and Miss Margaret F. Tattern, superintendent nurse of the Torquay Nurses' Home, has been appointed her successor.

### Lectures to Nurses on Anatomy.

By W. Johnson Smith, F.R.C.S., Principal Medical Officer, Seamens' Hospital, Greenwich.

### LECTURE V.—THE SPINE IN GENERAL.

The spine as a whole forms a firm and erect column well adapted to support heavy weights, and to protect very securely the important nerve structures it contains. Yet this column is not a rigid one, but is pliant and elastic so as to allow free bending and turning movements of the body, and to guard both brain and spinal cord against jarring from violent shocks.

The strength of the spine is due for the most part to the numerous ligaments which bind together its constituent bones; some passing along the whole length of the column

Fig. 10.—Vertical Section of three Vertebral Bodies, A, with Intervertebral Discs, B.

and others from vertebra to vertebra. Of the ligaments of the spine which are much varied in shape and direction, the most interesting and important are those interposed between the bodies of the vertebræ — the *intervertebral substances* or *discs* as they are called. Each of these (fig. 10) is a thick pad corresponding above and below in extent and shape to the corresponding surfaces of each pair of opposed vertebral bodies, and composed of an external zone of tough and firm fibrous or ligamentous tissue, and of a central mass of pulpy material which allows a certain degree of rocking movement of each vertebra, and by its elasticity suppresses undue concusssion. The external portion of each disc serves as a ligament to bind vertebral bodies together; the internal portion acts as a buffer.

The muscles are mostly arranged along the sides and back of the column forming a large and very intricate mass, made up partly of muscular structure or flesh, and partly of tendons or sinews, which extends from the pelvis to the skull and presents a perplexing variety of divisions and subdivisions, most of which are described by anatomists as distinct structures and supplied with distinguishing names. By the help of this large and complicated mass of flesh which almost completely removes from both sight and touch the posterior portion of the spine, man is enabled to maintain the upright position which constitutes one of his main anatomical characteristics. The part played by the numerous muscles attached to the spine in keeping it erect is indicated by the firm and steady attitude of a healthy adult which presents so marked a contrast with the bent back and uncertain gait of one enfeebled by age or disease.

The average length of an adult's spine from the atlas to the tip of coccyx is from 27 to 28 inches. If we exclude the sacrum and coccyx its length is about one-third that of the whole body. Owing to a slight yielding to pressure in the intervertebral substance, a person taking active exercise loses from one-third to half an inch in stature between breakfast and bedtime.

*The Curves of the Spine.*—In the new-born infant the spine as far as the sacrum is almost quite straight, or, rather, it forms a slight continuous curve with a posterior convexity (fig. 12). During childhood the curvature of the column becomes more marked and less simple, and a side view of the fully developed spine of an adult shows four alternating forward and backward curves: one forwards in the neck, the second backwards in the chest, another forwards in the lumbar region of the column, and the last backwards along the front of the sacrum (fig. 11). The third and lumbar curve, as has been pointed out by Professor Thomson, is one of the most remarkable features of the human spine, as it does not exist, or is developed to a slight extent only, in four-footed animals. There is also a normal lateral or side curve in the dorsal region which may be made out by tracing the long furrow in the skin which runs down the middle of the back, or by carrying a finger along the tips of the spinous processes which, it should be remembered, are the only portions of the vertebræ that can be readily felt in the living subject. The theory that this lateral deviation it due to more frequent use of one arm and to a consequent excess of muscular activity on the same side is favoured by the fact that the deviation is usually to be found on the right side.

Excessive and unnatural deviations of the spine presenting the different forms of spinal curvature, may occur as the results of disease or injury.

*Lateral Curvature* consists in extreme lateral deviation of the dorsal vertebræ associated with minor and compensating curves in other parts of the spine. This deformity affects most frequently ricketty children and weak "overgrown" girls, and may be due to a disturbance of the proper equilibrium of the body caused by an awkward position in reading or writing, or by loss or crippling of one of the lower limbs.

*Angular Curvature*, or a projection backwards of a portion of the spine, may be associated with lateral curvature, and due to like causes, but is usually the result of severe injury or of tuberculous disease. When as a result of fracture of the spine—"broken back"—one or more vertebral bodies are

Fig. 11. Fig. 12.

Diagrams Showing Curvature of the Spine in (fig. 11) the Adult and in (fig. 12) the Infant.

crushed or displaced, the bodies of the sound vertebræ immediately above and below the seat of injury fall together in front, and so cause a posterior projection of the spines beneath the skin of the back. A similar result would naturally follow softening and partial destruction of the vertebral bodies caused by disease.

In professional nomenclature the following terms are used to express the different forms of spinal curvature. 1. *Skoliosis*, lateral curvature; 2. *Kyphosis*, posterior or angular curvature; 3. *Lordosis*, anterior curvature; 4. *Pott's Curvature*, the variety of angular curvature caused by tuberculous or Pott's disease of the spine.

### District Nursing in Kimberley.

By a Nurse on the Spot.

Kimberley is no longer the place of interest it was two years ago when all eyes were turned in sympathy and admiration to applaud its gallant resistance of besieging Boers. That siege and the graphic accounts of it no longer appeal so powerfully to outsiders, but the time of stress and strain has left an indelible mark upon the place, and big and little graves in the quiet churchyard keep many memories green of that sad time. And there are those too, who in fancy can still hear the noise of fire-arms and see the white scared faces of the besieged, the wailing of starving little ones, and see the reeling gait of strong men, driven to drink, to drown, if possible, the craving for food and satisfy for a time, at least, the gnawing pangs of hunger. It is in our rounds as district nurses that we hear stories which bring home the reality of siege to us. More than one mother has told us, "This is my youngest now, nurse, but my baby died in the siege, there was no milk to be had." And again, "Yes, my poor husband died in the siege, he took fever, nurse, and he had eaten so little for weeks before that he couldn't battle with it." I could tell many sad stories, but I have headed my article "District Nursing" and not "Stories of the Siege," therefore I must not transgress.

### The Difficulties of the Work.

Our work is most interesting, but often very trying. I know nothing of district work at home and therefore cannot compare, but I should think the difference of climate must make such a work here rather more difficult; for instance, in midwifery cases, several hours spent in a room about 15 feet by 12 feet, with a temperature averaging 100°, is somewhat exhausting. Our patients are all sorts and conditions from jet black to purest pink and white. English, French, German, Dutch, Irish, Scotch, etc., and all religious denominations. Kimberley is a curious, rambling sort of town, the houses, each with an iron roof, are dotted down here, there, and everywhere; there is no regularity and no attempt at any symmetrical effect. You are in one street and out of it again before you realise that you have left the one you started in. This sounds complicated, but it is no more so than the streets, so I will not attempt to explain. The district is very much scattered, and extends for miles, so that we should be badly off if it were not that most of the district nurses possess bicycles. Fortunately, too, the various roads are passable for cyclists. Sometimes a call comes to us from some homestead standing out bare and lonely on the veldt, and one needs to be sure-footed to find a way at night where ant heaps and piles of débris give ample opportunities for tumbling ignominiously on one's nose!

### Jewish Families.

Our chief pleasure as district nurses is assuredly not in nursing in Jewish families, though they have afforded us much amusement. Their mode of living is decidedly novel; they have a curious habit of keeping all food supplies in their bedrooms. Here is a sketch of a shelf in a Jewish bedroom:—A pot of raspberry jam stands in close proximity to a tin of Keating's powder; next to the powder some biscuits, on which is resting the family toilet comb. Next some loaves of bread, on one of which leans lovingly a bottle of castor oil; next a package of sugar in which a family of flies is holding high revelry; and now the religious element is represented, and various books of prayer are found, on whose respected backs an open tin of sardines has found a resting place. Over all and through all dirt reigns supreme, and the Jew is well satisfied.

### Dutch Refugees.

Occasionally we are called to attend Dutch refugees. One house I went to interested me very much, and the patient acceptance of what seemed to be an exceedingly hard trial struck me as decidedly pathetic. The patient was a great clumsy Dutch woman, and at first sight decidedly uninteresting, but I learned to respect and admire her during the ten days I attended her. This was her first baby, and the little mite was born far away from home, in a bare, comfortless room, with glaring whitewashed walls and a calico ceiling; its little garments were neatly packed in a small wooden box, and though the mother left home in haste, bringing nothing but the bare necessaries for herself, not one thing had been forgotten for the comfort of the expected little one. No murmurs against an unkind fate, no bemoaning a hard lot, just a calm philosophy, which some might term stolidity, but which touched me more than I can say, and appealed to me as no whining, pitiful tale could ever have done.

### Little Real Poverty.

Some of the patients make us feel indignant, as their houses are really a disgrace, and water is not scarce in Kimberley; besides, there is remarkably little real poverty, and it is only a case of bad management or utter carelessness in many instances. Occasionally we come upon people who are really in want, and at such times we endeavour through the benevolent society to get them some help. As a rule I think the district nurses are very warmly welcomed, and it is a satisfaction to come away after an hour's work leaving cleanliness and comfort behind one. I think that we really achieve more in these visits than ensuring cleanliness and comfort. The work of trained and skilled gentlewomen done for these poor people has a refining influence; it may be the beginning of better things. Kimberley is not a pretty town, but to those who have lived here long and have seen it grow and thrive, it is better than any other part of the colony. The sunsets are its redeeming feature; even the huge débris heaps formed by waste material of years' accumulation from the mines are transformed and softened by the beautiful glow that comes to us at evening. Every tree and shrub stands out against the clear background, and the hardness of the iron-roofed houses is less pronounced, for one's whole thoughts are full of the glory of the sunset—now a bright orange, anon a lovely pink, which changes all too soon into palest mauve, and lastly a silvery haze, and the evening star peeps out a brilliant green in a lovely setting.

### Where to Go.

Concert to the Inmates of the British Home for Incurables, Crown Lane, Streatham, Saturday, November 30th, 6 p.m.

Sale of Work, National Orthopædic Hospital, Great Portland Street, Friday, November 29th and Saturday, November 30th, 2 to 6. To be opened on Friday by the Duchess of Marlborough.

Winter Sale of the Working Ladies' Guild, 24 Park Lane, by kind permission of Lord and Lady Brassey, on December 3rd, 4th, 5th. Princess Henry of Battenberg will open on December 3rd. 2 to 6 each day.

### To Nurses.

**We invite contributions from any of our readers, and shall be glad to pay for "Notes on News from the Nursing World," or for articles describing nursing experiences, or dealing with any nursing question from an original point of view. The minimum payment for contributions is 5s., but we welcome interesting contributions of a column, or a page, in length. It may be added that notices of entertainments, presentations, and deaths are not paid for, but, of course, we are always glad to receive them. All rejected manuscripts are returned in due course, and all payments for manuscripts used are made as early as possible after the beginning of each quarter.**

### Cæsarean Section.

The closing lecture of a Course on Obstetrics delivered to the nurses of the Glasgow Maternity Hospital on October 31st, 1901, by John M. Munro Kerr, M.B.C.M., F.F.P.S.(Glas.), Obstetric Physician, Maternity Hospital, Glasgow; Assistant to the Chair of Midwifery, Glasgow University, etc.

It occurred to me that, seeing this is the last lecture I will deliver to you this year, I might speak about some special subject connected with obstetrics. On thinking over the cases that have been in hospital under my care during the year, the large number of cases of Cæsarean section was most striking, and so I thought that perhaps you would like to hear something about this subject; for although few of you probably, after leaving here, will ever see the operation performed again, still it must be one that you will always remember with interest.

Within the last three months there have been five cases of Cæsarean section, and these, with the three I operated on in the early part of the year, make eight in all. They have all been operated upon because of contraction of the bony pelvis, and with few exceptions contraction of the pelvis is the indication for Cæsarean section. Sometimes we get tumours—tumours of the uterus, tumours of the pelvis—necessitating this operation, because they obstruct the vaginal passage; but such cases are very rare. The commonest indication, then, is deformity of the pelvis; and of the deformities of the pelvis, the rachitic is the one that we almost exclusively meet with.

The operation was performed in these eight cases; in two before labour had commenced, and in six after labour had started. At one time it used to be considered necessary that labour should have commenced before the operation was performed, but nowadays we do not consider that necessary; indeed, to operate before labour has commenced has two advantages—that preparations can be made more deliberately, and the time chosen for the operation.

As regards the preparation of the patients for the operation, we have them admitted to hospital, when possible, several days beforehand. Sometimes, of course, that cannot be arranged, as they only come to us when in labour. The patient then is kept in bed for two or three days, and the bowels are thoroughly evacuated once or twice. The skin, especially about the abdomen and vulva, is as thoroughly sterilised as we can make it. First of all we shave the hair from the pubis and any hairs round about the vulva and over the abdomen; we then scrub the abdomen and round about the vulva with soap and water, then successively with turpentine, alcohol, and 1 in 2,000 perchloride of mercury.

Finally, we put on a fomentation of 1 in 40 carbolic acid over the abdomen for 24 hours before the operation. Immediately before the operation the bladder is emptied by catheter, and the vagina is carefully cleansed, and the reason that now I take special precautions in cleansing the vagina is because in cases where sepsis occurs after the operation the infection comes usually from the vagina and not from the opening that is made in the abdomen. I take, then, very great care to thoroughly cleanse and sterilise the vagina. This is done by swabbing out the vagina, especially round about the cervix, with a 1 per cent. solution of lysol. The reason I do not use perchloride is because it causes the parts to shrink so much that it becomes difficult to swab them.

The instruments that are used for the operation are the ordinary instruments employed in abdominal operations, and they are sterilised by boiling. The sponges we use are sterilised swabs made of cotton-wool covered with fine gauze; these are made up in bundles of ten, and we take the precaution of boiling them again immediately before the operation, just in case of any accident having happened to them. One nurse attends to the sponges and wrings them out of hot sterilised water; another collects the soiled swabs, and is responsible for the right number being returned to her. The hands of the operator and his two assistants and nurses are cleansed in the same way as the skin of the patient, viz. by scrubbing in soap and water, then in turpentine, alcohol, perchloride, and lastly in carbolic. During the operation each washes his or her hands in a 1 in 40 solution of carbolic.

The operation is carried out by first making a longitudinal opening about 7 inches down the middle line of the abdomen. Having got into the abdominal cavity the operator has then to choose one of two methods: either to turn out the uterus first of all before he cuts into it, or simply to pack swabs round about the uterus and open it while it is inside the abdomen. The advantage of the former operation is that no fluid escapes into the abdominal cavity; the disadvantage, however, is that he requires to make a longer opening in the abdomen in order to let the uterus out, so that the advantages and disadvantages are about equally balanced. Having, then, chosen one of these methods, he proceeds to open up the uterus down the middle line in front. He does this in our hospital, as was first suggested by Professor Cameron, by putting a pessary (namely, a round piece of black vulcanite) over the distended uterus and pressing it firmly, then cutting inside of the ring till he comes down to the membrane. He then takes scissors and enlarges the opening to about 6 inches. That is the usual method adopted; but in the last two operations, the one on Monday night and the one on Tuesday morning, a different incision was made. An opening was made right over the fundus between the two tubes. There are certain advantages claimed for this incision. However, it is only on its trial at present, so we cannot say much about it.

The uterus, then, is got through by cutting on to the membranes. Having enlarged the opening to about six inches, one is able to extract the child, and the best way to do this is to seize hold of the feet. It is much more difficult to get hold of the head, as it is lower down usually; so the best way is to take the child out by the feet. A difficulty in the extraction is that sometimes one cuts down on the placenta, and you remember in the last case this happened, even though I made an incision over the fundus. In two of the other cases also I came down upon the placenta, so this occurs not infrequently. When that happens, the operator must get past the side of the placenta, or else pull out the placenta quickly and extract the child. Having got the child out, the next step is for the assistant to grasp the uterus firmly between his two hands, and compress it, to prevent any bleeding. At the same time the operator ties the cord and separates the child. The placenta and membranes are then expressed by the assistant, or removed by the operator introducing his hand into the uterus. He must take very great care to remove all the membranes. A portion of the membranes is very apt to be left behind, especially the part situated low down. Having removed the placenta and membranes, the assistant compresses the uterus firmly with hot swabs, and gets it to retract thoroughly. The uterus retracted, and still being firmly grasped by the assistant, it is then stitched, and I stitch with catgut. The catgut is known as "Hartman's Prepared Catgut," It is not used directly from the bottle, but is boiled for half an hour in absolute alcohol. The uterus is stitched up in such a way that the stitches go right through the wall of the uterus, except the mucous membrane. Usually about 12 to 15 stitches are put in.

Then they are carefully tied, and the uterus is completely sewn up. It is then thoroughly compressed, and any blood forced out through the cervix. After having stitched up the uterus it is very common to tie the fallopian tubes, so as to prevent the woman becoming again pregnant. This I have done in all the cases except two. In two it was not done, because the women were so exceptionally strong and healthy, and the results of Cæsarean section are so good nowadays that I thought it was right and wise to permit of them having perhaps another or two more children. In the other cases I tied and divided the tubes, chiefly because they were delicate women, and likely to run a great risk in undergoing another operation, especially one of them, who had bronchitis and heart disease.

The next step is to remove any blood clot that may have escaped into the abdomen, by swabbing the parts, especially behind the uterus, and in front between the uterus and the bladder. Having done that, the abdominal wound is closed, and this is done usually in three layers; the peritoneum is first stitched, then the muscular wall, and lastly the skin.

The whole operation takes 45 to 55 minutes. A great part of the time is taken up in carefully stitching the abdominal wound so as to prevent, if possible, the occurrence of hernia, because if one does not have good strong stitches the wound is apt to give and the bowels to protrude (ventral hernia); so we stitch up the wound in several layers with great care.

The dressing is then applied (double cyanide gauze), and it is not in the least necessary to dust the wound with any dusting powder. We just put a plain dressing over the wound, and cover it up with gauze and fix it in its place with some adhesive plaster, and then put a binder firmly round the abdomen. After that, to give the abdominal wall special support if there is much sickness, we often put on an elastic bandage.

As regards the after-treatment of these cases, there is nothing very peculiar to them as compared with other abdominal operations. I find it best to give the patients nothing for 24 hours—absolutely nothing for 24 hours. Sometimes, if they feel very well and only complain of thirst, little sips of hot water are given; but even that often seems to aggravate the sickness that is so liable to occur after chloroform. Another thing that sometimes is troublesome besides the sickness and the thirst is restlessness. For that I occasionally give $\frac{1}{6}$ gr. morphia hypodermically; but I try not to give morphia unless it is absolutely necessary, because morphia is a very undesirable medicine to give after abdominal section. It dulls the pain, but it cloaks over everything. If the patient has developed peritonitis, one does not become aware of it, because the morphia dulls the pain. It dulls all sensations and feelings; it also lessens the peristaltic action of the bowels, and so the patient gets into a dull, stupid condition, and sometimes one thinks she is going on all right when all the time there is some septic process at work. As far as possible, therefore, I avoid giving morphia.

As regards the special part of the nursing and sponging of the patient, that is done every few hours just after an ordinary labour. The parts are cleansed and carefully swabbed with perchloride or lysol solution, and a pad of sterilised absorbent cotton wool is placed over the vulva.

In none of the cases, except the one done on Tuesday, has there been any suspicion of fœtor in the lochia; but in that one it has to-day an unusually heavy odour, although she seems very well otherwise. If that continues I will douche out the vagina thoroughly, and if there should be evidence of any trouble in the uterus, I will douche out the uterus also with 1 in 2,000 perchloride. Usually by the fourth day after the operation we can tell whether the patient is going to recover or not, and after that day we allow greater liberties in the way of food, and so forth. After the first 24 hours I give them a little chicken tea; milk, I find, is not well digested by patients after abdominal section, so I prefer something in the way of chicken tea, which is a stimulant and contains a little nutriment. The bowels I have moved on the third or fourth day, and after that every second or third day as is deemed best. The abdominal wound I do not dress until the fourteenth day, when the stitches are removed and a fresh dressing is applied. In none of the cases has there been any trouble with the abdominal wound. The patient is kept in bed for four weeks at least, and then is allowed to sit up for a little. She is recommended to get an abdominal belt, and to wear it for some months. The mothers are able, as a rule, to commence to nurse their children by the sixth or seventh day.

That, briefly, is the course I follow with cases of Cæsarean section, and so far the results have been most satisfactory; for in the eight cases I have operated on, there has been no maternal death. Of course we cannot say much about these last two cases, because they are not yet "out of the wood," but the other six have all done very well, and I am hoping that it will be the same with them also.

These are very different results from what used to prevail, and the reason is that more care is taken, and so the possibility of septic mischief occurring is reduced to a minimum. Cæsarean section is not a difficult operation; it is not an operation attended with much risk to the patient, as there are seldom any complications. The results should be satisfactory, and they have become so now in the hands of the best operators.

Postscript.—November 19th. I am glad to say that the two cases last operated on have made a most satisfactory recovery. I should have mentioned in the lecture that all the children, except one, were extracted alive.

### Flowers for the Children's Ward.

By a Correspondent.

Surely one of the most pitiful sights is that of a little sick child, and many of us long to do something to lighten the burden of illness or convalescence, and bring a gleam of happiness into weary faces. Love of flowers is inherent in nearly all children, and the flower-decked tables in the wards are always a source of interest and delight, as are the bunches of fresh flowers which country dwellers forward sometimes to brighten the long hours of the children. But, alas! they last fresh such a short time, for as London air is full of smoke, smuts, and other kindred things, they naturally droop in a day or so at latest. Full of this regret an idea came into my mind as to whether it would not be possible for some bulbs to be grown in a simple way for the adornment of the rooms where the little sick children lie. It is quite practicable to grow many of the most charming spring flowers without any difficulty at all, and it would be well worth a little trouble to ensure the possession of flowers which would last some weeks, and give much pleasure to tired eyes which could daily note the change from the first appearance of bloom to the open flower. Any jar or bowl will do in which to grow the bulbs, and it is only needful to use just ordinary sand—it does not matter what kind. Presuming that the bulbs have been procured, get your bowls or jars one-quarter filled with sand; on this layer place the bulb and add more sand till they are half covered. Now place them in the dark, giving a little water, and keep the sand just damp. When the roots are fairly well grown and the green shoot of the bulb is just showing, bring them gradually into the light, and fill up the bowl with water, but do not let it cover the bulb. If planted early in December the flowers should be in bloom about the beginning of March, and they will last for weeks instead of days. The prettiest flowers to grow in this way are snowdrops, crocuses, trumpet daffodils, scyllas, jonquils, and the lovely little muscaris or grape hyacinths: these all flourish beautifully in sand and water. I believe that among the large growers many would give a few bulbs of this description if they were assured that they would be acceptable for the children's hospitals. There are always a quantity of bulbs left on their hands at the end of the season, and that is not too late for growing them in the way I have described. If the surplus specimens were applied for I do not think they would be refused. The bowls look very pretty with a carpet of green moss or canary seed, which can be planted when the bowl emerges from its retirement in the region of the dark cupboard. I think that any nurse trying this easy method of procuring bright and lasting flowers for her ward would not fail to please both herself and the little sick children under her care.

### Maternity Nursing Mission.

On Tuesday afternoon a meeting on behalf of the Maternity Nursing Mission, which has its headquarters at 5 Little James Street, Bedford Row, was held at the Passmore Edwards Settlement. Mr. H. C. Richards, M.P. for Finsbury, presided until another engagement obliged him to catch a train; and his place was then taken by Mr. Howard Barrett, Consulting Honorary Medical Officer to the Mission.

After a few preliminary remarks, the Chairman called upon Miss May, Matron and Hon. Secretary, to speak on the work of the Mission.

Miss May said that though this was the first time she had had to ask for money at a meeting, it was not the first time she had tried to impress people with the necessity of the proper nursing of the poor. As the work grew, new wants arose, and new developments took place; and thus, for almost the first time, the Union found itself in debt. The house in King's Cross Road had been given up partly because it was rather large, but chiefly because it was too expensive. At present the nurses were living partly over a stable, and they had their meals in the kitchen. They had none of the things now considered "modern requirements," and there was not even a bath-room. She could not quite say why they had got into debt; but she supposed that while people had been helping the brave fellows abroad, the Mission had been looking after their wives and widows at home. If 100 people would give £1 a year they could get on; but the anxiety was a great burden.

The Chairman drew attention to the fact that the debt on the Mission (£28) was a very small one, which ought to be easily cleared off in that room. The matron, he said, in spite of her cheerful manner, had not been paid the salary that was due to her; and he hoped that those present would help to get the nurses away from the stable as soon as possible.

Mrs. Humphry Ward said that the work of the Mission had grown out of a natural demand, and it was because of that demand that Miss May, who had been working in the neighbourhood as a nurse, put herself through midwifery training. She did not think the nurses who did such devoted work for the poor should have to put up with unnecessary hardships and privations. She remembered once coming across a woman (in Eastnor Place, not far away) who accosted the nurse with whom she (Mrs. Ward) was walking. She was neither clean nor sober. She said she was on the way to attend a confinement, and asked the nurse some simple question which even an untrained person could see belonged to the very A B C of nursing. The nurse declined to answer, and told her she had no right to ask such a question. The woman only grinned and went on. The circumstance had brought home to the speaker's mind the terrible risks to which the poor were exposed.

Mr. Wallace Bruce having given an outline of the Midwives Bill,

Mr. Howard Barrett, Hon. Consulting Medical Officer, said it might be asked why poor people did not call in the doctor for confinements. The poor had a wholesome dislike of the words "parish doctor"; and although his opinion of the medical officers under the Poor Law was very high indeed, he was glad that people should feel that dislike; he thought it tended to increase self-respect; and ordinary doctors' fees, although many put them very low, were too high for these women. What he liked about the Maternity Mission was that a payment was made by the patients. He knew, too, that Miss May was exceedingly particular as to what nurses she took on her staff. The Mission fell back on medical aid when necessary; and of this also he approved.

A vote of thanks to Mrs. Humphry Ward for lending the room closed the meeting.

### The Nurses' Bookshelf.

Lessons on Massage. By Margaret D. Palmer, Masseuse and Manager of the Massage Department of the London Hospital. (London: Baillière, Tindall and Cox. 1901. Price 7s. 6d. net.)

The tendency, for a long time back, has been for the public to regard massage as a great mystery and for the medical profession to make comparatively light of it. This has been due, no doubt, to a little ignorance on both sides. On the side of the public this goes without saying. But even in regard to medical men we cannot but feel that the light-hearted way in which massage is occasionally ordered, the extremely vague directions given, and the apparent indifference displayed in regard to the qualifications of those to whom the task is entrusted by some practitioners can only mean that they are not quite aware of the powerful influence for good or for evil which massage properly or improperly applied may exercise upon their patients. A point, however, has now been reached when practitioners really must acquire a certain degree of familiarity with the details of the process. The large extent to which certain forms of massage are now used in the treatment of fractures and the early stage of such injuries at which this treatment has to be applied, a stage so early that it would be out of the question to hand the cases over to any but the most skilful masseur, make it necessary that practitioners should not only know exactly what is to be done, but should be capable of themselves doing it if the necessity should arise. The book before us, however, is not intended for the medical student, but rather for the instruction of the ordinary masseuse. Indeed we cannot find that the treatment of fractures by massage is even alluded to, and certainly no directions are given as to the precautions necessary, and the manipulations permissible in dealing with these injuries after the methods which have recently come so much into vogue both here and on the Continent.

As an elementary handbook for the nurse who is being taught massage, this book will serve very well, for it is clearly written, and is evidently the outcome of a wide and extensive practical experience. Massage hinges so largely on anatomy that a very large portion of the book has had to be devoted to that sort of elementary anatomy which it is essential that the masseur or masseuse should be acquainted with, and all this is very well done. It is, however, to the part of the book which deals with its proper subject, namely massage itself, to which we turn with greatest interest, and here we find a very good description of the processes employed. First of all comes an account of the general technique, then the various regions are taken separately, and after their anatomy has been given the proceedings for the application of massage to the part in question are detailed; after this there are chapters on the application of massage to certain special regions and maladies, such as sprains, lawn-tennis arm, writers' cramp, spinal curvature, and general massage. An outline of the Weir-Mitchell treatment follows, and lastly various hints are given on the massage of children. Altogether a very useful, although not an absolutely complete, book. The illustrations are not only numerous, but some of them are distinctly above the average in artistic excellence.

### Everybody's Opinion.

[Correspondence on all subjects is invited, but we cannot in any way be responsible for the opinions expressed by our correspondents. No communication can be entertained if the name and address of the correspondent are not given as a guarantee of good faith, but not necessarily for publication. All correspondents should write on one side of the paper only.]

### THE NURSE'S NEVER.

"Matron" writes: In reference to the "Nurse's Never" I cannot refrain from saying that the protests from nurses which have appeared in these columns have been uncalled for and vulgar. I consider that the list which has appeared is valuable and that it has been put together after much consideration. It ought to appeal to every right thinking nurse. Trained nurses who have the supervision and guidance of nurses, both hospital, district, and private, will agree with me that even the best nurses require to be reminded of nursing rules. The perfect nurse has yet to be found, until then the "Nurse's Never" will be useful.

"R. B. N. A." writes: Although "Nurse Prudence" may have had "14 years' hospital and private experience" she has yet something to learn. When I was sister in a well-known hospital for paralysis and nervous diseases I was constantly asked by the resident physicians for the temperature of a patient half an hour after death, so that it became the rule of the ward that the nurses took the temperatures as a matter of course. The variations in the post-mortem temperatures were interesting to note.

### WHY ARE NIGHT NURSES NEGLECTED?

"Yorkshire" writes: I quite agree with everything "Matron" says with regard to the element of whining discontent which is slowly but surely creeping amongst nurses, and I for one am quite prepared to assert that present-day nurses are deteriorating. They lack the grit and stamina characteristic of former nurses, who sank self and "made the best of things." This grumbling about food, etc., is, I fear, not confined solely to night nurses, but is also becoming rife amongst day nurses. My experience of the matter is—the more refined type of nurse will endure hardships and the dreadful "plain living" without a murmur. But not so her coarser sisters; and there are a great, great number of the latter sort in the profession. Their coarseness and vulgarity must come out somewhere, consequently it breaks out in the form of grumbling and complaining about anything and everything. Whenever, by the by, I meet with a grumbler, my impression at once is, she comes from a very commonplace, vulgar home, and my surmise is usually correct. As things are going on, in 50 years hence, maybe sooner, a matron requiring nurses will insert in the advertisement "Good chef kept," or another matron wanting nurses will have in her advertisement "Epicures need not apply," and so on. We certainly are drifting in that direction.

### NURSING IN WESTERN AUSTRALIA.

"An English Nurse" writes: In your issue of August 10th there is a letter about my article on the above subject on May 18th. I should like to urge a few points of defence. Firstly, the article was not written in a bitter spirit, as I am not disappointed in this fair land, for the simple reason that it is my own country. Perhaps the editor headed "by an English nurse" because I was trained in an English hospital. It is that training which enables me to look at things from a broader point of view, and to realise that we do let things slide, and our nursing is behind the times. Secondly, if the "Westralian Nurse" had read the article more carefully she would have noted that nothing was said against trained nurses; on the contrary, I deplored the fact of the untrained woman acting nurse, and of the people being too easy-going to abolish her. I certainly did not mean to convey the curious idea that trained nurses let their typhoid patients bathe in a hip bath, etc. It was the want of nursing in these cases that appealed to me; but I stick to it that our surgical cases heal marvellously, and to the idea that the pure air has something to do with it. Thirdly, the principal hospitals are far from perfect, and if the Westralian nurse could but see, say, "Bart.'s," "St. Thomas's," or "King's," it would be a treat to observe astonishment and admiration. I am sadly afraid that she would be dissatisfied thereafter with her own. So far, they cannot be helped, for we are not rich yet and cannot afford large donations and contributions for their improvement and support. Meanwhile, therefore, we must work on and do our little best individually until the colony grows older, wiser, and more sensible, and takes us with her to help a great nation.

### NECESSARY AND UNNECESSARY WARD WORK AS TRAINING FOR PROBATIONERS.

"Nurse Ada" writes: I think most nurses may well thank you for publishing, and "Matron" for placing so accurately before your readers, the amount and difficulties of the unnecessary work of our training, and should "Matron's" letter be the starting point of a reform in this respect the sick public, as well as the nurses, may thank her too. How much a nurse's career in all its senses and phrases is coloured by the training school through which she passes only those who have finished their training, and feel their insufficiency of themselves to faithfully fulfil a nurse's duties and privileges, can gauge. I have just now finished a course of three years' training in one of our provincial hospitals. I did not enter hospital totally unitiated or utterly ignorant of the work that would fall to my lot; but looking back as I do now upon my training time I know the great "stress" upon myself and fellow nurses was not due to the "importunities of our patients, or to the over amount of our doctors' orders, nor to the intelligent carrying out of the same," but to the "unnecessary work." Unfortunately, this cleaning work is not confined to first or second year nurses, it extends throughout our training, and combined with the infinitely increased responsibilities of a third year nurse tends to make our last year's work almost appalling. One would not mind were our patients benefited—they are distinctly losers, for, accustomed or unaccustomed to manual labour as the nurse may be, her strength is most assuredly exerted upon the "unnecessary work" and not upon the "art of nursing." Beside this I have known many a nurse buy from her own slender wage extra polish or Monkey soap that she might bring her taps, etc., up to the standard expected of her; our scrubbing, indeed, was not confined to the ward furniture, it extended to the window sills outside the building. Now all arts have their rough as well as their smooth side, and has there not been ample proof in the past that we nurses "will" and "can" do the "dirtiest" as well as the "sweetest" part of our "art" if it will help in the humblest fashion to alleviate the physical or mental suffering of our fellow man or woman? You do not expect your boot boy at call to perform the rôle of "amusant" at your social functions, neither can it be expected that a nurse can intelligently and thoughtfully delicately handle or dress a wound or any other duty laid upon her by the doctor when the greater part of her time and strength has been spent in scrubbing and cleaning. How well I remember once when at the end of a very busy operating day Sister was "off" and I in charge for the time being. At seven o'clock, having still my centre furniture to scrub, a poor woman said to me, "Haint you orful tired, Nuss? I says to mysel' layin' 'ere, 'Ow does they do it? I wouldn't let my gal work like it, that I wouldn't." May I say one thing more? The effect of this "fag," from my own experience and observation of other nurses, is most demoralising. I have watched many a nice little pro. gradually getting disobliging, and impatient, only because the pressure all round of "unnecessary work," and much else that is more than unnecessary, is so great. I do not plead for myself personally; my training time is over. But I do plead for the women who are daily entering our hospitals to make themselves fit to stand in the presence of the "sick and dying."

"Sophia" writes: I read with great interest the article published in The Hospital for November 16th called "Necessary and Unnecessary Ward Work as Training for Probationers," by a late matron, and I most cordially agree with all she says. How truly she says that "a large portion of the probationer's time is taken up with domestic duties—sweeping, scrubbing of floors, lockers, tables, baths, etc.," the doing of which, to my mind, simply ruins a nurse's hands, but has no beneficial effect on her nursing powers whatever. Surely any probationer would find plenty of cleaning to do, as the writer suggests, in the operating theatre, cleaning lotion bowls, instruments, etc., which are closely connected with nursing. I think everyone will agree with me in saying that all nurses, more particularly surgical ones, should have smooth, soft hands. Is this at all possible when, at any rate for a year of her training, a nurse has to work like a charwoman? In a large provincial hospital where I was trained, one of our visiting surgeons told us one evening while lecturing, on no account to use the carbolic lotion, with which we disinfected our hands, too strong, as thereby we only made our hands very rough, and "a rough hand" said he "only harbours bacteria." A vision went through my mind at the moment of the probationers who spent a large portion of their time with their hands and arms up to their elbows in strong soda and water, scrubbing and cleaning, which was quite as likely to ruin their hands as 1-in-20 carbolic lotion. Concerning the "spirit of emulation among sisters" of which a "Late Matron" speaks, of course we all sympathise with them in liking a well-cleaned and polished ward, but some of them, particularly the elder ones, are rather fond of telling us how well they cleaned in their probationer days, forgetting that then there was no sterilising of instruments and dressings, besides many other things which are now absolutely indispensable, and that the nursing of patients then was much rougher than in the present day. Many sisters are apt to give the best reports to matron of nurses who scrub and clean the best, quite regardless of whether they are good and kind to their patients or not. I wish that all hospitals would adopt the excellent suggestion of "a few extra charwomen," as a "Late Matron" says. There would indeed be a higher standard of health in the nursing staff. I venture to think that many a poisoned finger is due far more to dirty work than touching dirty dressings. Frequently a nurse becomes anæmic and runs down, and perhaps has to give up her work altogether, simply because she is always *so* tired with all the cleaning she has to do. Then, again, the tired, worn nurse is by no means beneficial to the patients. One of the most important things in a nurse is that she should be bright and energetic, and how can she be this when she is tired to death with work that could quite well be done by some one else? Lastly, "A Late Matron" speaks of good nursing material being lost to the profession. I think we see such to be the case when we hear so many complaints about private nurses. In my experience of many years spent in private nursing I have so often heard complaints from private patients that nurses had been "so rough, so unrefined." Certainly housework does not tend to refine anyone, and many a refined woman, who would prove more valuable as a private nurse, is compelled to give up all thought of being a nurse because she is not strong enough to cope with the hard, rough work that she is obliged to do when she enters the hospital as a probationer.

### Workhouse Infirmary Nursing.

A meeting of the Sectional Committee appointed by the Executive Committee of the Workhouse Infirmary Nursing Association to consider Dr. Humphreys' scheme for the improvement of the nursing in Workhouse Infirmaries, was held last week. There were present:—Mr. W. Chance, Hon. Sec. Poor Law Conferences, in the chair; Miss Moir, Matron of St. Pancras Infirmary, Highgate; Miss Fynes-Clinton Miss Wilson, Treasurer, Workhouse Infirmary Nursing Association; Miss C. J. Wood, Mr. H. Bonham Carter, Dr. Humphreys, and Miss Gill, Secretary. After discussion, the feeling of the Sectional Committee was that it was undesirable at the present time to make any recommendations on Dr. Humphreys' scheme; but that it would be desirable to call a small meeting of guardians from rural Unions at the time of the Central Poor Law Conference in March, to place before them the difficulties in getting proper nursing in rural workhouses, and to ask for their assistance in removing these difficulties if possible. The following points to be specially considered:—(1) The friction between master and matron and nurse. (2) The payment of an adequate salary to the nurse. (3) The accommodation for the nurses and appliances for the sick. (4) The status of the nurse. (5) The appointment of women inspectors to Workhouses. It has been arranged for Dr. Humphreys to read a paper on his scheme at the Central Poor Law Conference in March, when an opportunity will be afforded of a full discussion by representative guardians.

### Appointments.

County Infirmary, Wexford.—Miss Mary Frances Pigott has been appointed nurse. She was trained at the City of Dublin Hospital, the Cork Street Fever Hospital, and the Royal Victoria Hospital, Bournemouth. She has also done private nursing.

Laurencekirk District Nursing Association.—Miss C. M. Nicol has been appointed nurse. She was trained at Leith General Hospital, and has since been staff nurse at Belvidere Fever Hospital, charge nurse at the Eastern, Northern, and Park Hospitals, London. She has lately, after doing private nursing in Bournemouth, been charge nurse at the Parochial Hospital, Dundee.

Malvern College Sanatorium.—Mrs. A. K. Süch has been appointed matron. She was trained at the Royal Hants County Hospital, Winchester, where she held the position of masseuse for some time, and subsequently she was sister of the men's medical ward.

The "Shaftesbury" Training Ship, Grays, Essex.—Miss Edith J. Wilson has been appointed nurse-matron. She was trained at the London Hospital, where she was afterwards for nearly three years staff nurse in the surgical ward. She has since, for two years, had charge of the infirmary attached to St. Nicholas Industrial School, Manor Park.

### Presentations.

Reading Union.—An interesting little ceremony took place in the Nurses' Home of the Reading Union Infirmary on the evening of the 22nd. The occasion was a social gathering to take farewell of Miss Else, who has been appointed superintendent nurse at the Staines Union Infirmary. Nurse Else, who has been in the service of the Reading Board of Guardians for five years, was trained at their Infirmary, where she obtained her certificate and was subsequently promoted to the position of charge nurse. She has also recently obtained the certificate of the London Obstetrical Society. As a memento of the esteem in which she was held by her fellow-workers, the nursing staff presented her with a polished oak writing cabinet, mounted with silver, and Superintendent Nurse Pinington, in presenting the gift, made a few appropriate remarks. Nurse Else also received parting gifts from the master and matron and other officials of the Union.

### Echoes from the Outside World.

AN OPEN LETTER TO A HOSPITAL NURSE.

Already the Coronation is more or less occupying everybody's thoughts, and on all sides fresh items of news, either authentic or founded upon desire, are retailed. The question of dress, though to the ordinary sightseer a matter of small importance—except that we shall all like to feel we look our best in honour of the occasion—is to those who will probably take part in some of the great pageants almost the first consideration. Queen Alexandra, of course, is obliged to devote a great deal of time and attention to this particular question, and her suggestions have up to the present given much satisfaction. English silks are being manufactured as quickly as it is possible to get them off the looms, for, since it is her Majesty's wish that home-made silks should be used as much as possible at the State functions, a wonderful impetus has been given to an industry which has already shown that it can hold its own in beauty of design and richness of make. At Spitalfields, Braintree, and Sudbury, several orders are being already executed for the Queen herself, in which gold is extensively employed, and the most magnificent cloth of gold will probably figure largely as the basis of the Royal Coronation robe itself. But it is not only in England that materials are being made for the coming festivities. In India many skilled workmen are busy executing the Royal orders for a quantity of the finest embroidery the production of which made Delhi, and, to some extent, Agra, famous in past times. This, it is believed, will be used principally for trimming or for the fronts of evening dresses; but, however it may be utilised, of course our dependencies beyond the seas are naturally pleased that they should thus share in the equipment of the Queen on such an auspicious occasion.

---

In addition to this a new State coach is to be built for the street procession, which is to take place the day after the Coronation. The superb coach which the King used at the opening of Parliament, though it is quite possible that it may be utilised in some way, is not suitable for an occasion when the atmosphere, it is hoped, will be warm, and the spectators will be numbered by thousands and tens of thousands, each hoping to obtain a glance of the Royal occupants. So an open carriage is being constructed for their Majesties, which will much resemble the full State landau which Queen Victoria used on the occasion of her Diamond Jubilee. It is to be lined with crimson satin, painted with the Royal Arms, etc., outside, and the vehicle is to be fitted with rubber tyres, a luxury not possible in State coaches. The State medal to commemorate the Coronation ceremony is already being arranged for, and it will be struck in two sizes. The larger will be made of gold, silver, and bronze. Also, it is said, that the fashion of wearing a particular flower on a particular day is to extend to the Coronation, and the blossom, chosen out of compliment to Queen Alexandra, is to be the lily of the valley, which, it is known, is her favourite flower. With the advantage of cold storage and forcing appliances it is possible nowadays to produce flowers, at almost any time of the year, in vast profusion; and if the cultivators have fair warning that millions of blooms will be required in June, they will be forthcoming. The selection of the lily of the valley seems wise, for the rose is already dedicated especially to St. George, and variety is charming even in flowers.

---

I was taken in to dinner the other evening by a young fellow whom I soon discovered had lately arrived from South Africa. He was a Colonial, and this was his first visit to our inclement clime, but, contrary to my expectation, he seemed to thoroughly enjoy the frost and the cold. My questions naturally drifted war-wards, and I elicited the fact, put in the most modest way, that he had been under arms for nine months last year and had fought right through to the relief of Ladysmith. Recent events prompted me to ask if he saw much of General Buller and I found that he had seen him daily during that time. When first the General came out the opinion in the Colonies was rather prevalent that he looked as if his life at home had been too luxurious for him to easily bear the hardships of the campaign, but this opinion rapidly vanished when business in real earnest began. Early in the morning, looking "fit" and well, he would mount his horse and ride off to more distant parts of the camp and was very soldier-like and alert. When a battle was going on directly a man galloped up with a message he bade him stand at once out of the way of the shot and shells although he himself remained where he was, an excellent target for the Boers. His personal bravery and his consideration for others endeared him to his men, and though my friend had no comment to make he was naturally grieved at the news of Sir Redvers' retirement from the service which greeted him when he landed here.

---

As to the part he played himself I had never before realised how hard it must have been to walk or ride all day in the blazing sun, and then go supperless to rest on the veldt, hoping for a meal next day. The narrator made no complaints, only urging that "things like that, you know, must be, At every famous victory," and that however good commissariat arrangements are they must occasionally fail, and the men starve for the time. He said that all new comers ate up their portion for the day during the twenty-four hours, relying on the next day's rations; the more experienced men always kept a bit of biscuit at the bottom of their pocket in case of emergency. He spoke in high terms of Government biscuits; they might be hard and tasteless he said, but they had a wonderful amount of nutriment in them in a small space. Many groans were heard when a pound of flour was given out instead of biscuits. Each man then tried his hand at cooking, but it was not an appetising cake made with no fat, no yeast, simply flour and water baked over a bit of tin on the top of a wood fire. The mixture was kept on the move as it cooked, lest it should stick, but when finished it was at best hard outside and of the consistency of dough inside, and at first produced maddening indigestion. After a time, this passed, but the return to biscuit was very welcome, except once when the men got to a town and found a Dutch girl willing to bake loaves at 6d. each, the soldiers providing the flour! Then they had a splendid meal! When food got very scarce, those with plenty of money willingly paid 3s. or 4s. for a single biscuit, and at other times the man who was beloved of his fellows was the one who had secured a bottle of curry powder so as to convert the stew of tinned beef and biscuit into a savoury mess.

---

It has wisely been decided not to proceed with the proposal to hold a "Day of National Humiliation." The Archbishop of York, in suggesting it, was, of course, animated by the highest of motives, but if his idea had been carried into effect many persons in this country would have been displeased, and erroneous conclusions would probably have been formed abroad. The memorial service at St. Paul's Cathedral on the afternoon of December 30th, which has been substituted, is not open to any objection, and there is no doubt that a large congregation will assemble out of respect to those who have fallen during the year in fighting against the enemies of England.

### For Reading to the Sick.

THE SEASON OF ADVENT.

In the first Advent God veiled his Divinity in flesh to prove the faithful; in the second Advent He will manifest His Glory to reward their faith.—*S. Chrysostom.*

Thou art coming ; we are waiting
With a hope that cannot fail,
Asking not the day or hour,
Resting on Thy Word of power,
Anchor'd safe within the Veil.
Time appointed may be long,
But the vision must be sure ;
Certainty shall make us strong,
Joyful patience can endure.

*F. R. Havergal.*

What of the Night ?
I would be patient—I will work and wait,
Thy stars are bright ;
But in the end, when watching hours grow late,
I pray not only, Lord, let there be Light—
" Let there be Night."

. . . . .

It is the *Day* ;
No more sad watchings by the midnight sea,
No twilight grey,
But, crown'd with light and immortality,
He stands from henceforth triumphing alway
In God's own Day. *B. M.*

Patient waiting and looking for the coming of Christ—the spirit of the man who is "looking for and hastning unto the Coming of the day of God;" is patiently waiting for the Advent of Jesus Christ. He may be still interested in everything here: in home, and children, and friends; in politics, and science, and art; in societies, and organisations, and all the outward mechanism of the Church. But he knows that the only "life" worthy of the name is that Eternal Life which is begun now, and which shall be fully manifested in that Day when the sign of the Son of Man shall be seen in the heavens.—*Bishop Wilkinson.*

Be now solicitous and sorrowful because of thy sins that at the Day of Judgment thou mayest be secure with the blessed. For then shall the righteous with great boldness stand against such as have vexed and oppressed them. Then shall the poor and humble have great confidence, but the proud man shall be compassed with fear on every side. Then shall He stand to judge them, who doth now humbly submit Himself to the censures of men.—*Thomas à Kempis.*

Grant, Lord, that we may thus live—though in perplexity, yet not in despair—bearing about in the body Thy dying; as men bereaved and earnestly seeking Thee, and ever looking for Thee, though it may be in feelings of desolation, yet not relaxing our diligence and watching, knowing this one thing only respecting Thy return, that it will come upon us all when we think not of it.

*Isaac Williams.*

It becomes us to celebrate with all devotion the advent of our Lord, being delighted with so much consolation, astonished at so much condescension, and warned by so much love. Nor think ye only of that advent in which He came to seek and to save the lost, but of that also in which He will come and take us to Himself. I would have you meditate deeply upon these two advents, pondering in your hearts how great things He did for us in the former, and what He hath promised us in the latter. For these are the two arms of the bridegroom, between which the bride sleepeth.

*St. Bernard.*

### Notes and Queries.

**The Editor is always willing to answer in this column, without any fee, all reasonable questions, as soon as possible.**
**But the following rules must be carefully observed:—**
**1. Every communication must be accompanied by the name and address of the writer.**
**2. The question must always bear upon nursing, directly or indirectly.**
**If an answer is required by letter a fee of half-a-crown must be enclosed with the note containing the inquiry.**

*Stamped Envelopes.*

Several correspondents have lately enclosed a stamped addressed envelope with their letter asking for information. We therefore think it necessary to state that the Queries addressed to us can only be answered in one of two modes, *i.e.*, either in the paper, in which case correspondents must wait their turn; or by letter, in which event, as we intimate every week, a fee of half-a crown must accompany the note containing the inquiry. It is absolutely useless to enclose stamped addressed envelopes, of which no notice can be taken.

*Uniform C.N.A.*

(87) 1. Will you kindly tell me what is the correct uniform for a nurse to wear on board ship? Should caps and aprons be worn? 2. Would you give me the address of the Colonial Nursing Association?—*Eva L.*

1. If you are not in charge of a patient, or if your patient does not object, wear ordinary dress. Let the requirements of the case be your guide. 2. The Imperial Institute, S.W.

*M. A. B.*

(88) I have noticed in advertisements in your columns "M. A. B. nurses need not apply," or "objected to." Can you tell me why? I have been told that the training at Hither Green was excellent. —*Lassie.*

It is impossible to know the private reasons for objecting to "special" training. One may conjecture, however, that it was because nurses trained in general hospitals were preferred.

*Massage.*

(89) I am thinking of taking a course of instruction from ——, but as work is not guaranteed to her pupils, I would like to know if there is any London association I could join, and to learn what I could earn in that particular branch. My wish is to live at home, and go to my patients' homes.—*L. S. C. and Nurse L.*

Write to the Secretary the Society of Trained Masseuses, 12 Buckingham Street, Strand, W.C.

*Soda Water: Cotton Wool.*

(90) 1. Is soda-water weakening to a patient with weak lungs? (2) Is it harmful to put cotton wool into the ears of a person suffering from a discharge from the same; and how often would you advise them to be syringed with water?—*Constant Reader.*

1. Possibly, but it is useful with milk, etc. Soda water often contains but little soda. 2. A patient with discharge from the ear must be placed under medical supervision without loss of time.

*Riviera.*

(91) I should be much obliged if you could mention any matron or institution through whom I could get a permanent engagement as private nurse on the Riviera.—*Nurse J. W.*

The most likely means of securing such a post would be through the Nice Nursing Institute, Villa Soleil, Avenue Thiers, Nice, or the Institute for Trained English Nurses, Via Roma, San Remo.

*New Zealand.*

(92) I am a private nurse, trained in New Zealand, with seven years' experience. I want to get about three months' nursing before I return, but it is difficult to find work. Please send me the last two copies of The Hospital, as perhaps some of the advertisements may suit me.—*E. B.*

You should advertise at once. Please send your order for copies of the paper to the manager.

**Standard Books of Reference.**

"The Nursing Profession: How and Where to Train." 2s. net; post free 2s. 4d.
" Burdett's Official Nursing Directory." 3s. net; post free, 3s. 4d.
" Burdett's Hospitals and Charities." 5s.
"The Nurses' Dictionary of Medical Terms." 2s.
" Burdett's Series of Nursing Text-Books." 1s. each.
"A Handbook for Nurses." (Illustrated). 5s.
" Nursing: Its Theory and Practice." New Edition. 3s. 6d.
" Helps in Sickness and to Health." Fifteenth Thousand. 5s.
"The Physiological Feeding of Infants." 1s.
"The Physiological Nursery Chart." 1s.; post free, 1s. 3d.
" Hospital Expenditure: The Commissariat." 2s. 6d.
All these are published by the Scientific Press, Ltd., and may be obtained through any bookseller or direct from the publishers 28 and 29 Southampton Street, London, W.C.

## Figures and Tables

**Fig. 10. f1:**
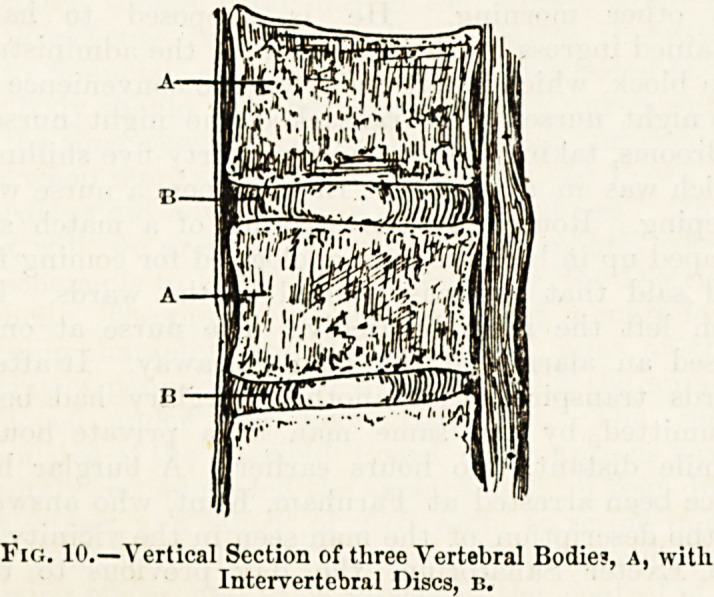


**Fig. 11. Fig. 12. f2:**